# Maternal Pre-Pregnancy Nutritional Status and Infant Birth Weight in Relation to 0–2 Year-Growth Trajectory and Adiposity in Term Chinese Newborns with Appropriate Birth Weight-for-Gestational Age

**DOI:** 10.3390/nu15051125

**Published:** 2023-02-23

**Authors:** Fengxiu Ouyang, Xiaobin Wang, Jonathan C. Wells, Xia Wang, Lixiao Shen, Jun Zhang

**Affiliations:** 1 Ministry of Education and Shanghai Key Laboratory of Children’s Environmental Health, Xinhua Hospital, Shanghai Jiao Tong University School of Medicine, 1665 Kong Jiang Road, Shanghai 200092, China; 2 Center on the Early Life Origins of Disease, Department of Population, Family and Reproductive Health, Johns Hopkins University Bloomberg School of Public Health, Johns Hopkins University School of Medicine, Baltimore, MD 21205, USA; 3 Childhood Nutrition Research Centre, Population, Policy and Practice Research and Teaching Department, University College London, Great Ormond Street Institute of Child Health, London WC1N 1EH, UK

**Keywords:** maternal obesity, gestational weight gain, gestational diabetes, birthweight for gestational age, growth trajectory, adiposity

## Abstract

Being born with appropriate weight-for-gestational age (AGA, ~80% of newborns) is often considered as low risk for future obesity. This study examined differential growth trajectories in the first two years by considering pre- and peri-natal factors among term-born AGA infants. We prospectively investigated 647 AGA infants and their mothers enrolled during 2012–2013 in Shanghai, China, and obtained repeated anthropometric measures at ages 42 days, 3, 6, 9, and 18 months from postnatal care records, and onsite measurements at age 1 and 2 years (skinfold thickness, mid-upper arm circumference (MUAC)). Birthweight was classified into sex-and gestational age-specific tertiles. Among mothers, 16.3% were overweight/obese (OWO), and 46.2% had excessive gestational weight gain (GWG). The combination of maternal prepregnancy OWO and high birthweight tertile identified a subset of AGA infants with 4.1 mm higher skinfold thickness (95% CI 2.2–5.9), 1.3 cm higher MUAC (0.8–1.7), and 0.89 units higher weight-for-length z-score (0.54, 1.24) at 2 years of age with adjustment for covariates. Excessive GWG was associated with higher child adiposity measures at 2 years of age. AGA infants manifested differential growth trajectories by the combination of maternal OWO and higher birthweight, suggesting that additional attention is needed for those “at increased risk” of OWO in early intervention.

## 1. Introduction

The first 1000 days of life (from conception to age 24 months) represents a critical period for developing lifelong metabolic health, as well as an optimal window for early-life obesity intervention [1]. In utero exposure to maternal adverse conditions has permanent programming effects [2,3,4]. Both small and large for gestational age (SGA, and LGA) infants have been linked to an increased risk of later cardiometabolic diseases [5,6]. In contrast, infants born with appropriate birth weight-for-gestational age (AGA), consisting of ~80% of newborns, are often considered as conferring a low risk for future obesity and are, thus, overlooked in obesity prevention [7]. An unsettled clinical question is whether factors assessed prenatally could help to refine infant obesity risk assessment at postnatal care for AGA infants during early childhood. There is limited evidence exploring this topic.

It has been recognized that maternal prenatal nutritional characteristics including maternal prepregnancy obesity, excessive gestational weight gain (GWG) and gestational diabetes mellitus (GDM) are risk factors for high birthweight and child obesity [8,9], while inadequate GWG is associated with an increased risk of infant SGA [9,10]. Women with obesity and excess GWG are commonly identified as being at increased risk of GDM and infant LGA [11]. However, these maternal prenatal factors are seldom taken into account by pediatricians in the risk assessment of obesity for infants, and postnatal child care is relatively separate from maternal prenatal care in the typical clinical model. Fetal exposure to the “in utero” unfavored maternal nutritional environment has permanent programming effects on later obesity and adult cardio-metabolic diseases [2,3,4]. In fact, the developmental origins of health and disease (DOHaD) approach has been adopted in the early intervention of childhood obesity at ongoing trials, for example, the Healthy Life Trajectories Initiative (HeLTI) project [12]. The intervention was adapted to individual risk status assessed in each phase from preconception to prenatal, postnatal, infancy and childhood [7,12]. A clinical question raised is whether pre-pregnancy/prenatal maternal nutritional status should be considered in the identification of newborns at increased risk of future obesity among AGA infants during early childhood.

This study aimed to examine whether there is a differential growth trajectory in the first two years and a heterogeneity of obesity risk by considering pre- and peri-natal factors among AGA infants, which can be an opportunity for early risk assessment and obesity prevention. As preterm birth infants may have very different growth trajectories [13], this study was limited to term-born children.

## 2. Methods

### 2.1. Study Population

This study used data from the Shanghai Obesity and Allergy Birth Cohort Study. The primary objective of this birth cohort study was to examine early life environmental exposure and maternal risk factors of childhood obesity and allergic diseases. The baseline study was conducted between June 2012 and February 2013 at two large tertiary hospitals in Shanghai, China. Pregnant women were recruited when they were admitted to our study hospitals for deliveries. Eligibility criteria included: (1) having routine prenatal care at the study hospitals; (2) singleton pregnancy; (3) planning to stay in Shanghai for the next 2 years; and (4) a willingness to participate in this study and sign the consent form. After enrollment, trained study nurses conducted a face-to-face maternal questionnaire interview to collect information including prepregnancy weight, education, and smoking and passive smoking during pregnancy, etc. The women normally gave birth to their babies in the next 1–2 days after our investigation. After mothers delivered their babies and before their discharge from the hospital, our study nurses reviewed their medical records to abstract data including prenatal care, laboratory reports, pregnancy complications, labor and delivery course, and birth outcomes (gestational age, infant sex, birth weight, and birth length). We aimed to recruit 500 women in each of the two hospitals, with a total sample of 1000 pregnant women. At the end of February 2013, a total of 1243 women were eligible and enrolled. Among them, 829 of the children had a postnatal follow-up visit. The 23 children born preterm (gestational age <37 weeks) were excluded from this report (Figure S1).

We followed the mother-child pairs using a web-based questionnaire investigation at the age of 6 months, and invited them for follow-up visits at Xinhua Hospital at age 1 and 2 years, during June 2014 and April 2015. The follow-up visit at 2 years of age included a face-to-face questionnaire interview of the mother, and a physical examination and blood draw of the child. We obtained signed informed consent from all mothers. The study was approved by the Institutional Review Boards of Xinhua Hospital and the International Peace Maternity and Child Hospital.

### 2.2. Maternal Prenatal Nutritional Factors

Prepregnancy weight was self-reported by women at the baseline investigation. Maternal height, GDM, the last weight before delivery, and the mode of delivery were abstracted from hospital medical records.

Maternal prepregnancy BMI (kg/m^2^) was calculated as prepregnancy weight (kg)/height squared (m^2^), and categorized as: underweight BMI < 18.5 kg/m^2^, normal weight 18.5–23.9 kg/m^2^, overweight 24–28 kg/m^2^ and obese ≥ 28 kg/m^2^, based on the Chinese BMI classification standards for the adult population [14].

GWG was defined as the difference in maternal weight between prepregnancy weight and the last measure before delivery. The last measure of maternal weight was usually performed at the time of hospital admission, about 1–2 days before delivery. As we described previously, GWG was categorized as excessive, appropriate, or insufficient based on GWG above or below the recommended IOM 2009 guidelines without adjustment and according to prepregnancy BMI: 12.5–18 kg (BMI < 18.5 kg/m^2^); 11.5–16 kg (BMI 18.5–24.9 kg/m^2^); 7–11.5 kg (BMI 25–29.9 kg/m^2^); and 5–9 kg (BMI ≥ 30 kg/m^2^) [9,15,16].

The diagnosis of GDM followed the recommendation of the International Association of Diabetes and Pregnancy Study Groups (IADPSG) [17,18]. Specifically, a 75-g oral glucose tolerance test (OGTT) was performed at 24–28 weeks of gestation on all pregnant women. GDM was defined if any of the following plasma glucose values reached: (1) fasting: ≥5.1 mmol/L; (2) 1 h: ≥10.0 mmol/L; and (3) 2 h: ≥8.5 mmol/L.

### 2.3. AGA Definition and Sex- and Gestational Age-Specific Birthweight Tertiles among AGA

Infant birthweight, gestational age, and sex were abstracted from hospital medical records. The gestational age at delivery was estimated by using maternal last menstrual period (LMP), with supporting information from early ultrasound measures (<20 weeks). If the gestational age estimated by ultrasound measures differed by >7 days from that by LMP, then we used the ultrasound assessment.

Birthweight-for-gestational age status was categorized as LGA, AGA, or SGA based on Chinese references for birthweight at each gestational week in boys and girls [19]. LGA was defined as birthweight >90th percentile, AGA as the 10th–90th percentile, and SGA as <10th percentile, respectively. Among AGA infants, gestational age-specific birthweight was then classified into low, medium and high tertile in boys and girls, respectively. Among 806 term born children, 647 were born AGA. This report used data from the 0–2 years of 647 term born AGA infants.

### 2.4. Child Anthropometric Measures

At age 12 and 24 months, weight, length, mid-upper arm circumference (MUAC), and skinfold thickness were measured by study nurses according to the WHO protocol, and described elsewhere [20]. With 70–90% of total adipose tissue located subcutaneously, skinfold thickness can be used to reflect total body fat [21].

We also prospectively obtained repeated anthropometric measures (weight and length) at ages 42 days, 3, 6, 9, and 18 months from postnatal care records, which were measured by trained nurses according to the WHO protocol at community health care centers in Shanghai.

Sex-specific z-scores weight-for-length (ZWFL) were calculated using WHO Child Growth Standards (WHO 2006). Z-score = (observed value—median value of WHO growth standards)/standard deviation (SD) of the WHO growth standard (http://www.who.int/childgrowth/standards/en/; accessed on 10 February 2023) [22].

### 2.5. Postnatal Covariates

Infant feeding type (formula feeding, exclusive breastfeeding, and mixed breastfeeding) at age 0–6 months was obtained based on the mother’s report at the postnatal 6 months online survey. Child exposure to passive smoking (yes, no) during age 0–24 months was obtained based on a questionnaire interview at each postnatal follow-up at 6, 12 and 24 months, and it constituted exposure to passive smoking if the mother reported a “yes” at any of the follow-up time points.

### 2.6. Data Analysis and Statistics

**Figure 1:**
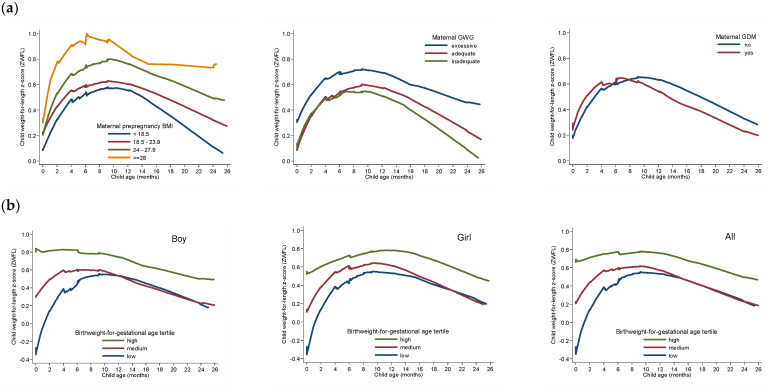
LOWESS regression plot of child weight-for-length z-score (ZWFL) at age 0–2 years in children born appropriate for gestational age (AGA) by (**a**) maternal prepregnancy BMI categories, GWG and GDM status; and (**b**) child birthweight for gestational age tertiles (low, medium, and high) in the total sample and in boys and girls, respectively.

**Figure 2:**
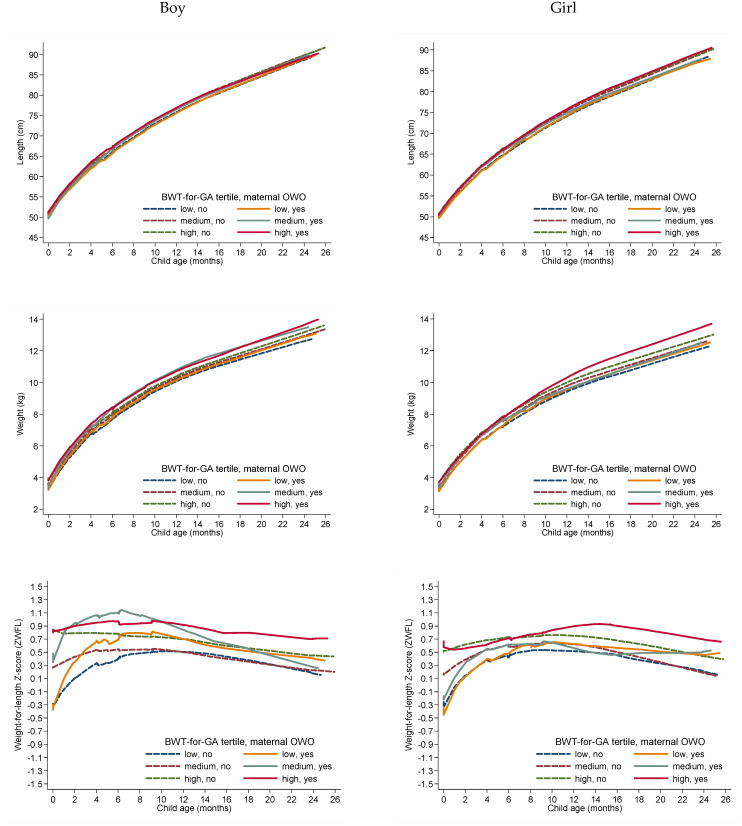
Lowess regression plot of length, weight and ZWFL from age 0–25 months by the sex- and gestational age-specific tertiles of birthweight and maternal prepregnancy OWO in 674 term-born AGA children (354 boys and 320 girls). OWO: overweight and obese; AGA: appropriate birth weight-for-gestational age; BWT for GA: birth weight-for-gestational age.

This study examined the combined association of maternal pre-pregnancy nutritional status and infant birth weight-for-gestational age tertiles (low, medium, and high) with child age 0–2 year-growth trajectory and adiposity measures in term-born Chinese children. Among AGA infants, we present ZWFL (the index used to define OWO by WHO standards) from age 0–2 years (a) by prenatal factors (prepregnancy BMI categories, GWG, and GDM status, respectively); and (b) by infant birthweight for gestational age tertiles (low, medium, and high) in boys, girls, and the combination of both, respectively, using locally weighted smoothing (LOWESS) plots (Figure 1). We also used a Spaghetti plot and LOWESS plots to present length, weight and ZWFL across 0–2 years by the six groups of maternal OWO status (yes, no) and birth weight-for-gestational age tertiles (low, medium, and high) combinations in boys and girls (Figure S2 and Figure 2). To evaluate these associations, generalized estimating equation (GEE) linear models were used with an exchangeable correlation structure assumed to accommodate repeated postnatal outcomes (child anthropometric adiposity measures) during 0–6 months, and 7–25 months, respectively.

Multivariate linear regression models were then used to examine the association of pre- and perinatal factors with child anthropometric adiposity measures at 2 years of age. All regression models included the same covariates, which included mode of delivery, infant age, sex, feeding type at age 0–6 months (formula feeding, exclusive breastfeeding, and mixed breastfeeding), maternal passive smoking during pregnancy (yes, no), and child passive smoking (yes, no). The approach to the selection of covariates for the regression models was to control for potential confounders and gain estimate precision. All analyses were performed using SAS 9.2 software (SAS Institute, Cary, North Carolina) and STATA 15.1 (Corp, College Station, TX, USA).

## 3. Results

Of 806 term newborns, 12.4% were LGA, and 4.0% were SGA (Figure S1). This study focused on 674 AGA infants. The mean maternal age was 29.4 years (SD 3.5 years) at childbirth. Mean prepregnancy BMI was 21.2 kg/m^2^ (SD 3.0 kg/m^2^). The proportion of maternal prepregnancy overweight was 13.2%, obesity 3.1%, and underweight 17.8%. During pregnancy, 46.2% had excessive GWG, 15.4% had inadequate GWG, and 12.5% (*n* = 84) had GDM. Gestational age ranged 37–41 weeks (Table 1 and Table S1).

### 3.1. Longitudinal Data Analysis of Birthweight-for-Gestational Age Tertiles, Prenatal Factors and Adiposity Measures among AGA Infants at Age 0–2 Years

As shown by Figure 1, Table 2 and Figure S2, AGA infants vary in their growth trajectory and adiposity, depending on maternal characteristics. AGA infants in the high birthweight tertile had higher ZWFL at age 0–2 years compared to those in the low tertile (Figure 1). Newborns in the top tertile of birthweight-for-gestational age and with maternal OWO had the highest weight (β = 0.87; 95% CI: 0.45–1.29 kg) and ZWFL (0.62; 0.31–0.93 unit) at 7 to 25 months with adjustment for mode of delivery, infant age, sex, feeding type at age 0–6 months, maternal passive smoking during pregnancy, and child passive smoking (both *p* < 0.001, Table 2, Figure 2).

In AGA infants, children of mothers with excessive GWG had on average a 0.19 (0.08, 0.31) unit higher ZWFL at age 0–6 months, and a 0.18 (0.04, 0.31) unit higher ZWFL at 7–25 months than those with adequate GWG with adjustment for the same covariates as in Table 2. GDM was not associated with ZWFL among children born AGA in this study (Supplemental Table S2).

### 3.2. Prenatal Factors, Birthweight-for-Gestational Age Tertiles, and Body Composition among AGA Infants at 2 Years of Age

In the follow-up onsite visit at 2 years of age, skinfold thickness and MUAC were measured. Among AGA babies, the combination of a high tertile birthweight-for-gestation and maternal OWO identified a subset of infants with 4.1 mm higher skinfold thickness (95% CI 2.2–5.9), 1.3 cm higher MUAC (0.8–1.7), and 0.89 units higher ZWFL (0.54, 1.24) at 2 years of age compared to those with low tertile birthweight and without maternal OWO after the adjustment for covariates (Table 3).

As shown in Table S3, excessive GWG was associated with higher ZWFL, skinfold thickness, and MUAC in children at age 2 years with adjustment for covariates, but the magnitudes of these associations were lesser than maternal OWO related associations. GDM was not associated with child adiposity measures among children born AGA in this study.

## 4. Discussion

This prospective cohort study examined differential growth trajectories in the first two years by considering pre- and peri-natal factors among newborns of AGA, which is novel.

We found that AGA infants with a combination of high birthweight tertile and maternal OWO identified a subset of the elevated adiposity among AGA infants. In addition, excessive GWG was associated with higher values in all adiposity measures in AGA children at 2 years of age.

Previous studies reported that AGA neonates with reduced antenatal/fetal growth velocity exhibited significant postnatal catch-up growth [23]. Our results in AGA infants with low tertile of birthweight-for-gestational age is in agreement with previous findings [23]. Of note, within the AGA group, a combination of high birthweight tertile and maternal OWO was strongly associated with elevated adiposity values at age 2 years with adjustment for covariates. In this study, the definition of maternal overweight (BMI 24–28) and obese (BMI > 28) were based on the Chinese adult BMI cut points, which has been found to be associated with an increased risk of non-communicable diseases (NCDs) including hypertension, diabetes, and dyslipidemia in the adult population [24,25]. GWG was categorized based on IOM 2009 guidelines [16]. The results suggest that AGA infants with maternal OWO and excessive GWG were still at increased risk of obesity despite their “normal” birth weight, and should receive additional attention regarding obesity prevention/interventions at postnatal child care visits. This proposal can be tested in our ongoing randomized trial [12].

The research question addressed by this study was raised as a result of observations during clinical practice. In current practice, AGA term-born infants are often considered at low risk of future obesity and are thus overlooked for obesity intervention efforts at postnatal care visits. This is also due to a gap between obstetric/maternity and pediatric/child care at the transition point from the pre- and perinatal to the postnatal phase in clinical practice. The present study revealed that even for AGA term-born infants, modifiable maternal prenatal risk factors of child obesity including maternal OWO and GWG are not only intervention target points before conception and during pregnancy, but also biomarkers of risk factors and predictors of child obesity. In Shanghai, children at age 0–3 years had standard postnatal care (including growth monitoring) according to established Shanghai child healthcare working protocols, which were provided by nurses and pediatricians from local Community Healthcare Centers. Low birthweight/SGA and infant overweight/obesity are identified as “high risk” for additional attention and consultation. By identifying predisposing prenatal factors of obesity among AGA infants, it can inform evidence-based practice in the development of early interventions to prevent obesity among children “at increased risk” of obesity and reinforce postnatal intervention. Future risk model analysis will allow us to estimate the childhood overweight and obesity risks associated with the individual predictor variables in a larger cohort study.

Cumulative evidence suggests that childhood obesity and its related cardiometabolic risk factors should have their roots in the pre-pregnancy and/or pregnancy period [7,26]. Evidence from this study supported the hypothesis that obesity prevention should start prior to conception and concurrently address multiple prenatal risk factors of adverse birth outcomes [1,7,27]. A recent meta-analysis reported that maternal pre-pregnancy BMI and GWG were associated with risk for GDM, LGA and SGA, and GWG associations with the adverse outcomes assessed were to a lesser degree [28]. Maternal prepregnancy OWO and GWG should both be considered as targets for the prevention of high or low birthweight [29]. In this study, we focused on AGA infants and their mothers. Maternal underweight can be associated with increased risk of SGA and lower values in child BMI and ZWFL at the age of 2 years. The positive associations between maternal BMI and infant birthweight [30] and child adiposity might be due to both a genetic contribution and shared family environment conditions (dietary patterns, physical activity, sedentary lifestyle, screen time, etc.) [31,32].

In contrast, GDM was not associated with child adiposity measures at 2 years of age among term-born AGA infants in this study. This might be partly attributable to the routine screening and timely treatment of GDM in prenatal care in Shanghai, one of the most developed areas in China. A recent meta-analysis also revealed that the association between GDM and the risk of child OWO attenuated towards the null after additional adjustment for maternal BMI, suggesting that it was largely explained by maternal BMI [33].

This prospective study has important strengths. We collected high quality clinical data on multiple pre-, peri- and postnatal risk factors, and repeated anthropometric measures and skinfold thickness in young children. All women received routine prenatal care at the study hospitals, and women with GDM received treatment at the time of diagnosis. In this study population, the initial prenatal care was usually registered at about 6–14 gestational weeks. The routine prenatal care included the monitoring of maternal GWG and screening for gestational diabetes at 24–28 gestational weeks, etc. Caution is needed related to the generalizability of our finding on GDM to other populations. This study also had limitations. Prepregnancy weight was self-reported by pregnant women at the baseline investigation. However, at the population level, the judgements about excessive, adequate or inadequate GWG in this study should be able to reflect overall categories upon current available guidelines and tools. Any measurement errors associated with weight at conception, GWG and compliance with IOM 2009 guidelines, if it existed, would be unlikely to increase measures of association. Instead, the bias was more likely to hide associations that might exist toward a finding of no association. A future cohort study starting from preconception or at early pregnancy, with weighing scales calibrated in study clinics or using specific study scales by mothers, might harvest more precise measurements for prepregnancy BMI and GWG. Furthermore, this report only examined children up to 2 years of age; long-term follow-up of these children would allow us to explore the long-term health impacts of prenatal factors and birthweight.

In summary, AGA infants manifested differential growth trajectories by the combination of birthweight for gestational age and maternal BMI. This finding underscores that AGA term born infants, who represent the majority of newborns, are heterogenous in their future risk of OWO and present opportunities for early obesity intervention.

## Figures and Tables

**Table 1:** Maternal and child characteristics in 674 term-born AGA children.

Maternal Characteristics	Mean ± SD, or *n* (%)	Infant Characteristics	Mean ± SD, or *n* (%)
Maternal age at childbirth (years)	29.4 ± 3.5	Gestational age (weeks)	39.0 ± 1.0
Prepregnancy BMI (kg/m^2^)	21.2 ± 3.0	Birthweight (g)	3359.8 ± 281.5
Mother Education		Infant sex	
High school or lower	85 (12.6)	Boy	354 (52.5)
College or above	589 (87.4)	Girl	320 (47.5)
Mother passive smoke during pregnancy		Feeding Type (0–6 months)	
Yes	196 (29.2)	Formula feeding	73 (12.7)
No	475 (70.8)	Exclusive Breastfeeding	204 (35.5)
Maternal prepregnancy BMI (kg/m^2^) categories		Mixed breastfeeding	297 (51.7)
<18.5	120 (17.8)	Children passive smoking	
18.5–23.9	443 (65.8)	No	317 (48.2)
24–27.9	89 (13.2)	Yes	341 (51.8)
>28	21 (3.1)	Child anthropometric measures at 2 years (*n* = 451)	
Gestational Weight Gain		Child age, months	23.8 ± 0.5
Adequate	256 (38.4)	Length	88.8 ± 3.0
Excessive	308 (46.2)	Weight	12.8 ± 1.5
Inadequate	103 (15.4)	BMI (kg/m^2^)	16.2 ± 1.4
GDM		Weight for length z-score (ZWFL)	0.37 ± 0.96
no	588 (87.5)	BMI for age z-score	0.37 ± 0.98
yes	84 (12.5)	Length for age z-score	0.58 ± 0.94
Mode of Delivery		Weight for age z-score	0.60 ± 0.91
Vaginal delivery	185 (27.4)	Sum of skinfold thickness (mm)	22.8 ± 5.1
C-Section	489 (72.6)	Mid-Upper Arm Circumference (MUAC) (cm)	15.8 ± 1.2

**Table 2:** Longitudinal data analysis of the associations of gestational age-specific tertiles of birthweight and maternal prepregnancy overweight or obesity (OWO) with child anthropometric measures in 674 term-born AGA children from birth to age 6 months (*n* = 2731 measures), and from age 7 to ~25 months (*n* = 2579 measures).

		0–6 Months		7–25 Months	
Maternal OWO	BWT-for-GA Tertile	β (95% CI)	*p*	β (95% CI)	*p*
		Length (cm)			
No	1st (low)	Ref.		Ref.	
	2nd (medium)	0.63 (0.38, 0.88)	<0.00001	1.15 (0.71, 1.58)	<0.00001
	3rd (high)	1.02 (0.75, 1.28)	<0.00001	1.18 (0.70, 1.66)	<0.00001
Yes	1st (low)	−0.02 (−0.47, 0.43)	0.94	−0.09 (−0.864, 0.693)	0.83
	2nd (medium)	0.52 (0.04, 0.99)	0.03	0.71 (−0.103, 1.527)	0.09
	3rd (high)	1.00 (0.62, 1.38)	<0.00001	1.04 (0.33, 1.74)	0.004
		Weight (kg)			
Maternal OWO	BWT-for-GA tertile				
No	1st (low)	Ref.		Ref.	
	2nd (medium)	0.26 (0.17, 0.35)	<0.00001	0.30 (0.11, 0.50)	0.003
	3rd (high)	0.48 (0.39, 0.58)	<0.00001	0.49 (0.28, 0.69)	<0.00001
Yes	1st (low)	0.13 (−0.01, 0.28)	0.07	0.11 (−0.22, 0.45)	0.52
	2nd (medium)	0.40 (0.21, 0.58)	0.00004	0.39 (−0.10, 0.88)	0.12
	3rd (high)	0.48 (0.32, 0.63)	<0.00001	0.87 (0.45, 1.29)	<0.0001
		Weight-for-length z-score (ZWFL)			
Maternal OWO	BWT-for-GA tertile				
No	1st (low)	Ref.		Ref.	
	2nd (medium)	0.29 (0.16, 0.43)	<0.0001	0.05 (−0.11, 0.21)	0.50
	3rd (high)	0.56 (0.42, 0.71)	<0.0001	0.24 (0.08, 0.39)	0.003
Yes	1st (low)	0.21 (−0.02, 0.45)	0.08	0.14 (−0.11, 0.40)	0.27
	2nd (medium)	0.52 (0.30, 0.74)	<0.0001	0.21 (−0.13, 0.56)	0.23
	3rd (high)	0.55 (0.35, 0.75)	<0.0001	0.62 (0.31, 0.93)	<0.0001

Generalized estimating equation (GEE) linear models were used to accommodate repeated postnatal anthropometric measures. All models were adjusted for mode of delivery, infant age, sex, feeding type at age 0–6 months (formula feeding, exclusive breastfeeding, and mixed breastfeeding), maternal passive smoking during pregnancy (yes, no), and child passive smoking (yes, no).

**Table 3:** The association between gestational age-specific tertiles of birthweight (BWT-for-GA tertile) and maternal prepregnancy overweight or obesity (maternal OWO) and child anthropometric measures of body composition in term AGA children at 2 years of age.

		Child Anthropometric Measures at Age 2 Years in AGA Children						
		*n*	Mean ± SD	β (95% CI)	*p*	Mean ± SD	β (95% CI)	*p*
Maternal OWO	BWT-for-GA tertile		Sum of skinfold thickness (mm)			Mid-Upper Arm Circumference (MUAC) (cm)		
No	1st (low)	133	22.8 ± 4.4	Ref.		15.7 ± 1.1	Ref.	
	2nd (medium)	128	21.8 ± 4.6	−1.1 (−2.2, 0.1)	0.08	15.7 ± 1.1	−0.01 (−0.3, 0.3)	0.95
	3rd (high)	116	22.2 ± 4.3	−0.6 (−1.8, 0.6)	0.37	15.8 ± 1.1	0.1 (−0.2, 0.4)	0.34
Yes	1st (low)	19	24.0 ± 3.7	1.5 (−0.9, 3.8)	0.22	15.8 ± 1	0.1 (−0.5, 0.6)	0.75
	2nd (medium)	16	25.9 ± 6.7	3.1 (0.6, 5.6)	0.01	16.4 ± 1.4	0.7 (0.1, 1.3)	0.02
	3rd (high)	34	26.7 ± 8.4	4.1 (2.2, 5.9)	<0.0001	17.0 ± 2.0	1.3 (0.8, 1.7)	<0.0001
			Weight (kg)			Weight-for-length z-score (ZWFL)		
Maternal OWO	BWT-for-GA tertile							
No	1st (low)	133	12.3 ± 1.3	Ref.		0.19 ± 0.84	Ref.	
	2nd (medium)	130	12.7 ± 1.3	0.4 (0.06, 0.7)	0.02	0.22 ± 0.97	0.03 (−0.20, 0.25)	0.82
	3rd (high)	118	13.0 ± 1.3	0.7 (0.4, 1.0)	<0.0001	0.46 ± 0.82	0.27 (0.04, 0.50)	0.02
Yes	1st (low)	19	12.6 ± 1.3	0.3 (−0.4, 1.0)	0.36	0.43 ± 0.76	0.25 (−0.20, 0.70)	0.28
	2nd (medium)	16	13.3 ± 1.9	1.1 (0.4, 1.8)	0.003	0.84 ± 0.97	0.65 (0.17, 1.14)	0.01
	3rd (high)	34	13.9 ± 2.3	1.5 (1.0, 2.0)	<0.0001	1.09 ± 1.42	0.89 (0.54, 1.24)	<0.0001

BWT-for-GA tertile: sex- and gestational age-specific tertiles of birthweight 1st (low), 2nd (medium), and 3rd (high) tertiles; maternal OWO (prepregnancy BMI > 24 kg/m^2^) (yes, no). Multivariate linear regression models were used and adjusted for mode of delivery, infant age, sex, feeding type at age 0–6 months (formula feeding, exclusive breastfeeding, and mixed breastfeeding), maternal passive smoking during pregnancy (yes, no), and child passive smoking (yes, no).

## Data Availability

Data is not readily available because access to the deidentified participant research data must be approved by the research ethics board on a case-by-case basis. Please contact the corresponding author F. Ouyang for assistance in data access request.

## Supplementary Materials

The following supporting information can be downloaded at: https://www.mdpi.com/article/10.3390/nu15051125/s1, Figure S1: Participant flow diagram; Figure S2: Spaghetti plot of 5310 longitudinal anthropometric adiposity measures from age 0–25 months and lowess regression plot by the sex- and gestational age-specific tertiles of birthweight and maternal prepregnancy overweight in 674 term-born AGA children (354 boys and 320 girls); Table S1: Birthweight distribution in grams for each of gestational age-specific birthweight tertiles (low, medium, and high) among boys and girls born appropriate for gestational age (AGA); Table S2: Longitudinal data analysis of the association between maternal prepregnancy BMI categories, gestational weight gain, gestational diabetes mellitus (GDM) and child weight-for-length z-score in 674 AGA infants at birth to age 6 months (*n* = 2731 measures) and from age 7 to ~24 months (*n* = 2579 measures); Table S3: Association between maternal prepregnancy BMI categories, gestational weight gain (GWG), gestational diabetes mellitus (GDM) and child anthropometric measures of body composition at age 2 years.

## Abbreviations

LGA: large for gestational age; AGA, appropriate weight for gestational age; SGA, small for gestational age; OWO, overweight and obesity; GWG, gestational weight gain; GDM, gestational diabetes mellitus; BMI, body mass index; MUAC, mid-upper arm circumference; ZWFL: weight-for-length z-score; DOHaD, the developmental origins of health and disease; SD, standard deviation.
