# Supplementary material for: Maternal Pre-Pregnancy Nutritional Status and Infant Birth Weight in Relation to 0–2 Year-Growth Trajectory and Adiposity in Term Chinese Newborns with Appropriate Birth Weight-for-Gestational Age
Source: Nutrients. 2023 Feb 23;15(5):1125. doi: 10.3390/nu15051125 (PMC10005412; doi:10.3390/nu15051125)
Supplement: Supplementary file 1 [file nutrients-15-01125-s001.zip › nutrients-2126971-supplementary.pdf]

Figure S1. participant flow diagram

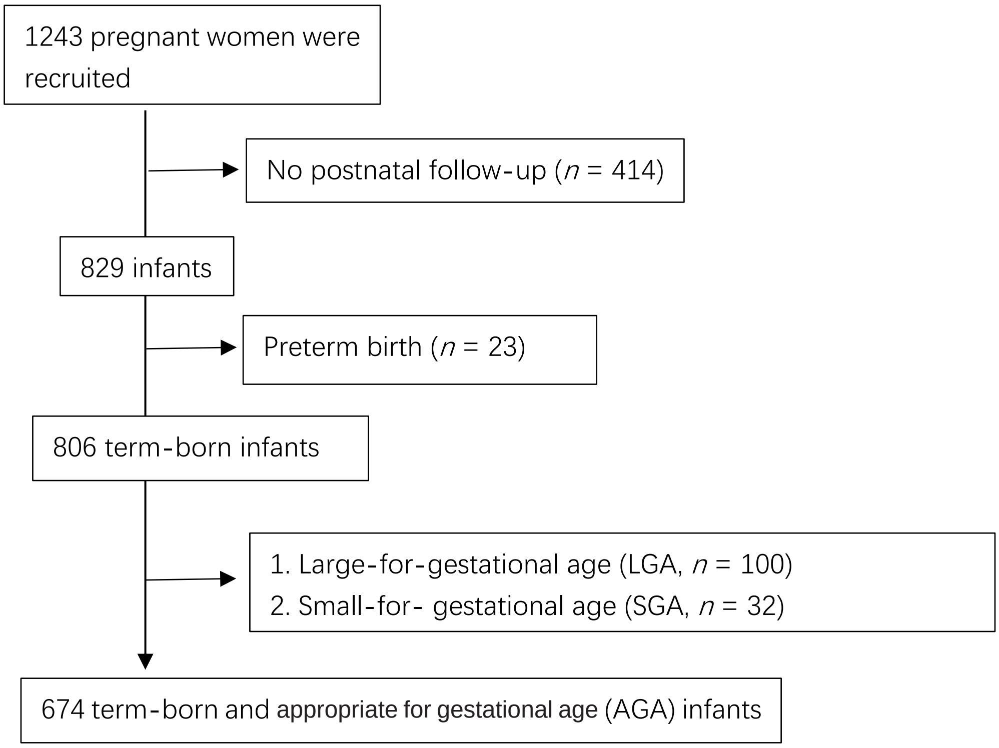

Table S1. Birthweight distribution in grams for each of gestational age-specific birthweight tertiles (low, medium, and high) among boys and girls born appropriate for gestational age (AGA).

	Gestational age-specific birthweight tertiles among AGA infants											
Gestational age (weeks)	Low				Medium				High			
	n	Minimum	maximum	Mean (SD)	n	min	max	Mean (SD)	n	min	max	Mean (SD)
Boy												
37-	6	2655	2800	2721(48)	7	2830	3070	2935(97)	7	3190	3600	3397(153)
38-	32	2780	3200	3052(107)	31	3210	3470	3314(78)	30	3480	3790	3657(77)
39-	47	2880	3275	3107(111)	50	3300	3560	3424(83)	49	3570	3940	3746(108)
40-	22	2960	3330	3192(101)	23	3345	3640	3490(101)	22	3660	4030	3821(103)
41 weeks+ 0-6 days	9	3145	3460	3313(101)	10	3485	3685	3572(72)	9	3745	4005	3876(84)
Girl												
37-	4	2590	3050	2826(188)	5	3060	3300	3183(107)	5	3310	3420	3377(47)
38-	30	2705	3060	2928(99)	30	3070	3350	3209(93)	30	3370	3670	3483(92)
39-	43	2815	3210	3072(103)	44	3215	3445	3316(66)	43	3450	3800	3609(107)
40-	22	2920	3300	3132(122)	22	3330	3500	3414(65)	22	3525	3870	3648(107)
41 weeks+ 0-6 days	6	2930	3275	3061(126)	7	3295	3460	3384(53)	7	3470	3870	3684(124)

Figure S2. Spaghetti plot of 5310 longitudinal anthropometric adiposity measures from age 0-25 months and lowess regression plot by the sex- and gestational age-specific tertiles of birthweight and maternal prepregnancy overweight in 674 term-born AGA children (354 boys and 320 girls).

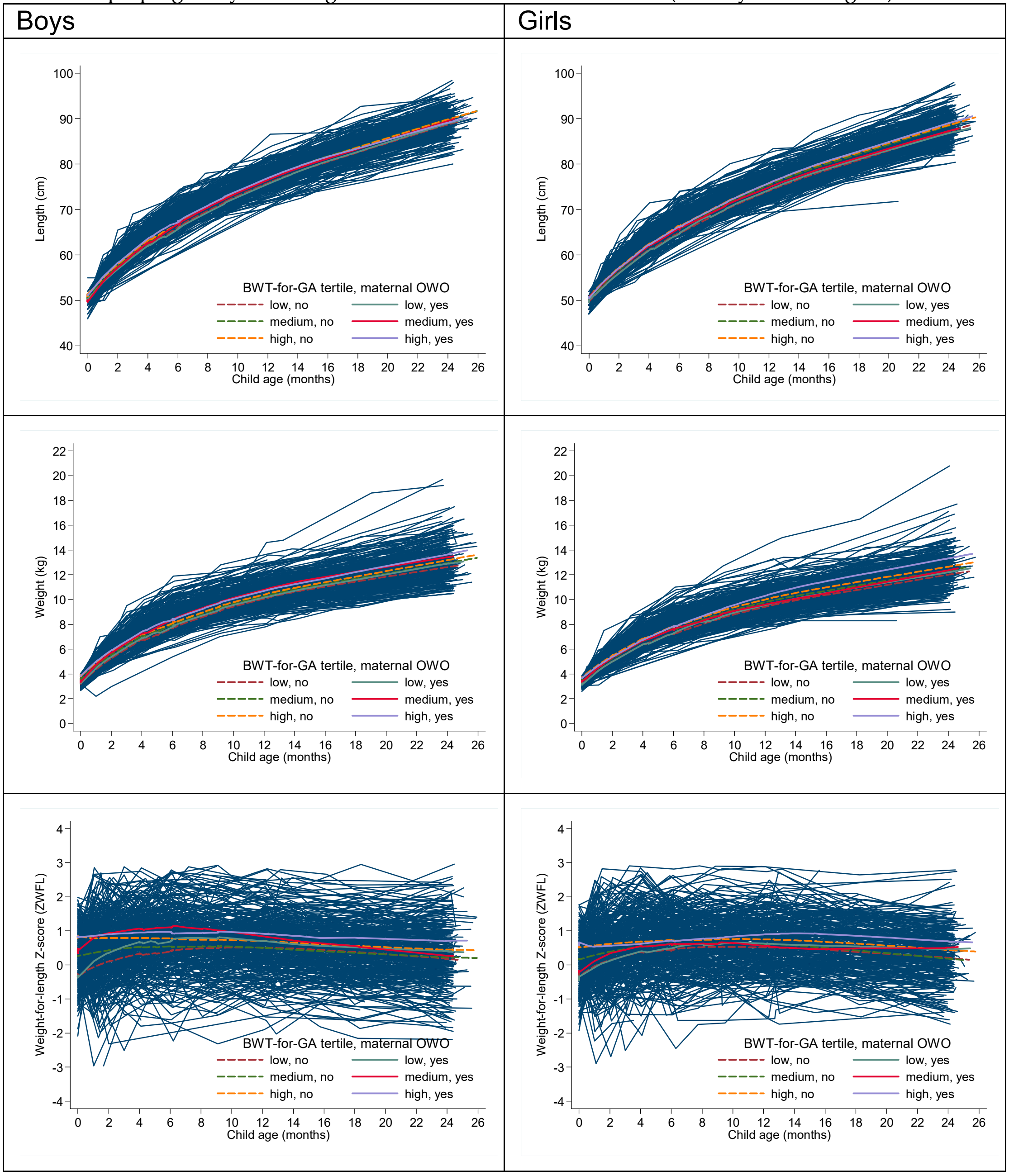

BWT-for-GA tertile: sex- and gestational age-specific tertiles of birthweight 1st (low), 2nd (median), and 3rd (high) tertiles; maternal OWO (prepregnancy BMI > 24 kg/m2; yes, no).

Table S2. Longitudinal data analysis of the association between maternal prepregnancy BMI categories, gestational weight gain, gestational diabetes mellitus (GDM) and child weight-for-length z-score in 674 AGA infants at birth to age 6 months (*n* = 2731 measures) and from age 7 to ~24 months (*n* = 2579 measures).

Predictors	**Weight-for-length z-score (ZWFL) Among AGA infants**			
	**0-6 months**		**>=7 months**	
	β (95% CI)	p	β (95% CI)	p
Maternal prepregnancy BMI (kg/m^2) categories				
<18.5	-0.09 (-0.22,0.05)	0.21	-0.10 (-0.25,0.06)	0.24
18.5-23.9	Ref.		Ref.	
$\geq$ 24	0.14 (0.02,0.27)	0.03	0.26 (0.07,0.44)	0.007
Gestational Weight Gain				
Adequate	Ref.		Ref.	
Excessive	0.19 (0.08,0.31)	0.001	0.18 (0.04,0.31)	0.009
Inadequate	0.002 (-0.15,0.15)	0.98	-0.11 (-0.28,0.07)	0.25
GDM				
No	Ref.		Ref.	
Yes	0.07 (-0.08,0.22)	0.38	-0.05 (-0.26,0.17)	0.68

Generalized estimating equation (GEE) linear models were used to accommodate repeated postnatal anthropometric measures. All the models were adjusted for maternal passive smoking during pregnancy, mode of delivery, infant sex, child age, feeding type at age 0-6 months, and child passive smoking.

Table S3. Association between maternal prepregnancy BMI categories, gestational weight gain (GWG), gestational diabetes mellitus (GDM) and child anthropometric measures of body composition at age 2 years.

		Child Anthropometric measures at age 2 years in AGA infants					
	N	mean±SD	β (95% CI)	p	mean±SD	β (95% CI)	p
		**Length (cm)**			**Weight (kg)**		
Prepregnancy BMI (kg/m²)							
<18.5	79	88.8±3.0	0.22(-0.51,0.94)	0.56	12.4±1.2	-0.26(-0.62,0.09)	0.14
18.5-23.9	301	88.7±3.0	Ref.		12.7±1.4	Ref.	
24-27.9	53	88.9±3.4	-0.01(-0.86,0.85)	0.99	13.3±2.1	0.53(0.12,0.95)	0.01
> 28	16	89.3±2.3	0.69(-0.78,2.17)	0.36	13.8±1.9	1.12(0.41,1.84)	0.002
GWG							
Adequate	168	88.8±2.9	Ref.		12.7±1.3	Ref.	
Excessive	201	88.8±3.2	-0.03(-0.63,0.58)	0.93	13.0±1.7	0.28(-0.01,0.58)	0.06
Inadequate	77	88.7±2.8	-0.15(-0.93,0.63)	0.70	12.4±1.1	-0.34(-0.73,0.04)	0.08
GDM							
No	395	88.8±3.0	Ref.		12.8±1.4	Ref.	
Yes	55	88.6±3.1	-0.33(-1.16,0.50)	0.43	12.7±1.9	-0.07(-0.48,0.34)	0.72
		**BMI (kg/m²)**			**Weight-for-length z-score (ZWFL)**		
Prepregnancy BMI (kg/m²)							
<18.5	79	15.8±1.1	-0.43(-0.76,-0.10)	0.01	0.05±0.85	-0.29(-0.52,-0.06)	0.01
18.5-23.9	301	16.2±1.3	Ref.		0.34±0.89	Ref.	
24-27.9	53	16.8±1.6	0.61(0.22,0.99)	0.002	0.76±1.13	0.42(0.14,0.69)	0.003
> 28	16	17.3±2.3	1.14(0.47,1.81)	0.001	1.14±1.39	0.82(0.35,1.29)	0.001
GWG							
Adequate	168	16.1±1.3	Ref.		0.30±0.89	Ref.	
Excessive	201	16.5±1.5	0.35(0.08,0.63)	0.01	0.55±1.03	0.24(0.05,0.44)	0.02
Inadequate	77	15.8±1.0	-0.38(-0.74,-0.02)	0.04	0.03±0.78	-0.27(-0.53,-0.02)	0.03
GDM							
No	395	16.2±1.3	Ref.		0.37±0.93	Ref.	
Yes	55	16.2±1.8	-0.02(-0.41,0.37)	0.92	0.36±1.18	-0.02(-0.29,0.25)	0.89
		**Sum of skinfold thickness (mm)**			**Mid-Upper Arm Circumference (MUAC) (cm)**		
Prepregnancy BMI (kg/m²)							
<18.5	78	21.4±4.2	-1.06(-2.27,0.15)	0.09	15.6±0.9	-0.2(-0.50,0.09)	0.17
18.5-23.9	297	22.5±4.5	Ref.		15.8±1.1	Ref.	
24-27.9	53	25.7±7.5	3.42(1.99,4.84)	<0.0001	16.5±1.7	0.76(0.42,1.11)	<0.0001
> 28	16	26.1±5.4	3.59(1.14,6.05)	0.004	16.4±1.9	0.68(0.08,1.27)	0.03
GWG							
Adequate	167	22.2±4.8	Ref.		15.7±1.2	Ref.	
Excessive	196	23.7±5.5	1.44(0.40,2.49)	0.007	16.1±1.3	0.38(0.14,0.63)	0.003
Inadequate	78	22.0±4.2	-0.12(-1.46,1.22)	0.86	15.6±1.1	-0.09(-0.40,0.23)	0.60
GDM							
No	390	22.7±4.7	Ref.		15.9±1.1	Ref.	
Yes	55	23.5±7.1	0.81(-0.62,2.24)	0.27	15.8±1.7	-0.12(-0.46,0.22)	0.49

Multivariate linear regression models were used, and adjusted for mode of delivery, infant age, sex, feeding type at age 0-6 months (formula feeding, exclusive breastfeeding, and mixed breastfeeding), maternal passive smoking during pregnancy (yes, no), and child passive smoking (yes, no).
